# Endemic lizard *Gallotia galloti* is a paratenic host of invasive *Angiostrongylus cantonensis* in Tenerife, Spain

**DOI:** 10.1017/S0031182022000336

**Published:** 2022-06

**Authors:** Lucia Anettová, Elena Izquierdo-Rodriguez, Pilar Foronda, Vojtech Baláž, Ladislav Novotný, David Modrý

**Affiliations:** 1 Department of Botany and Zoology, Faculty of Science, Masaryk University, Brno, Czech Republic; 2 Instituto Universitario de Enfermedades Tropicales y Salud Pública de Canarias, Universidad de La Laguna, La Laguna, Canary Islands, Spain; 3 Department of Obstetrics and Gynecology, Pediatrics, Preventive Medicine and Public Health, Toxicology, Legal and Forensic Medicine and Parasitology, Universidad de La Laguna, La Laguna, Canary Islands, Spain; 4 Department of Ecology and Diseases of Zoo Animals, Game, Fish and Bees, Faculty of Veterinary Hygiene and Ecology, University of Veterinary Sciences Brno, Palackého tř. 1946/1, 612 42 Brno, Czech Republic; 5 Institute of Parasitology, Biology Center of Czech Academy of Sciences, České Budějovice, Czech Republic; 6 Finn Pathologists, CVS Group plc, Norfolk, UK; 7 Novopath Ltd, Ceperka, Czech Republic; 8 Department of Veterinary Sciences, Faculty of Agrobiology, Food and Natural Resources/CINeZ, Czech University of Life Sciences Prague, Prague, Czech Republic

**Keywords:** *Angiostrongylus cantonensis*, *Gallotia galloti*, invasive nematode, paratenic host

## Abstract

*Angiostrongylus cantonensis* is an invasive zoonotic nematode which causes eosinophilic meningitis in accidental hosts – vertebrates including humans – and is known to impact wildlife. Even though the parasite originates in Southeast Asia, it has spread worldwide, especially into fragile island ecosystems. The Canary Islands are a biodiversity hot spot with numerous endemic species affected by biological invasions. Among others, *Rattus rattus* and *Rattus norvegicus* threaten many endemic species by predation and may spread invasive pathogens, such as the rat lungworm *A. cantonensis*, which was first described in Tenerife in 2010. Since it is known that lizards can act as paratenic hosts for *A. cantonensis* and *Gallotia galloti* is a lizard abundant in Tenerife, the aim of this study was to reveal the role of these endemic lizards in the parasite's life cycle. *Gallotia galloti* were captured in Tegueste, Tenerife, and after euthanasia, liver and tail muscle samples were examined for the presence of *A. cantonensis*. During microscopic examination, 8/36 liver samples (22.2%) contained granulomas with nematode larvae. In total, 10/39 liver samples (25.6%) and 7/36 tail muscle samples (19.4%) were positive for *A. cantonensis* DNA using qPCR. This is a first report of a reptile endemic to the Canary Islands acting as paratenic host of *A. cantonensis*. The fact that the parasite is obviously well-established in the island ecosystem and exploits endemic lizards as hosts may have important implications for the parasite's ecoepidemiology. Moreover, the parasite might threaten other species which depend on lizards in the island trophic web.

## Graphical abstract

**Figure:**
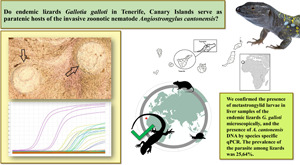

## Introduction

*Angiostrongylus cantonensis*, the rat lungworm, is an invasive metastrongylid nematode associated with species of the Rattini, which was recently confirmed in the island of Tenerife (Foronda *et al*., 2010). Currently, the parasite is well established in the humid northern areas of the island (Martín-Carrillo *et al*., 2021). This nematode is known to be a generalist when it comes to paratenic hosts, i.e. its third-stage larvae, which are infective for the definitive, paratenic or accidental hosts, has been described in fish, amphibians and saurians (Wallace and Rosen, 1967; Ash, 1968; Radomyos *et al*., 1994). Additionally, several invertebrate species such as planarians or centipedes have been proven to be paratenic or transport hosts of the parasite (Wang *et al*., 2018; Chaisiri *et al*., 2019). The paratenic hosts have the capacity to accumulate infective larvae and serve as an infection source to avian and mammalian hosts that suffer from neurological disorders like eosinophilic meningitis (Wallace and Rosen, 1967; Alicata, 1991; Paredes-Esquivel *et al*., 2019).

In Tenerife, *Rattus rattus* and *R. norvegicus* are definitive hosts of the parasite. The rats have been present in the Canary Islands approximately since the 15th century and occupy practically all habitats where, together with cats, they are the major invasive predators (Nogales *et al*., 2006). The impact of rats in Tenerife's ecosystem not only includes the predation of vertebrates (like lizards), which is probably the biggest negative consequence of their presence, but also to a lesser extent negative effect on invertebrates such as gastropods. Besides the impact of predation, the invasive rodents are an important source of various pathogens (Foronda *et al*., 2011; Abreu-Yanes *et al*., 2018), including zoonotic nematodes, such as *A. cantonensis*. Three species of molluscs have been confirmed as intermediate hosts of *A. cantonensis* in Tenerife: *Cornu aspersum*, *Theba pisana* and *Plutonia lamarckii* (Martin-Alonso *et al*., 2015).

The terrestrial fauna of Macaronesian archipelago is dominated by reptiles that colonized the islands drifting from the coast of North Africa and further specialized through processes of adaptive radiation (López-Jurado and Mateo, 1995; Cox *et al*., 2010). Three saurian genera – *Gallotia*, *Chalcides* and *Tarentola* of the families Lacertidae, Scincidae and Gekkonidae – inhabit the islands; however, the terrestrial *Gallotia* spp. reach fairly highest densities and play an important role in Macaronesian ecosystems (Valido and Nogales, 1994; Molina-Borja and Bischoff, 1998). In Southeast and East Asia, *A. cantonensis*, saurians are known to be paratenic hosts of *A. cantonensis* and the consumption of their raw organs or meat by humans has been associated with eosinophilic meningitis outbreaks (Radomyos *et al*., 1994; Hidelaratchi *et al*., 2005). Similarly, lizards are known to be paratenic hosts in the life cycle of metastrongylids of carnivores (Jeżewski *et al*., 2013). The abundance of lizards in hyperendemic areas of *A. cantonensis* in Tenerife, together with the diet of these saurians, makes the encounters between *G. galloti* and infective L3 larvae of *A. cantonensis* highly probable. The aim of the study was to determine the possible involvement of *G. galloti* in the life cycle of *A. cantonensis* in Tenerife.

## Materials and methods

### Samples collection and the microscopy

**Fig. 1.**
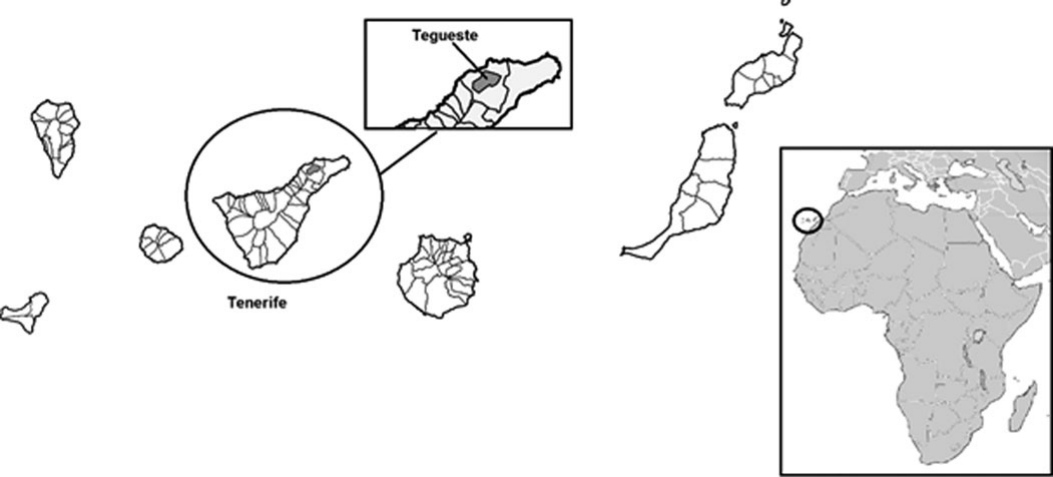
Sampling area, Tenerife, Canary Islands.

Thirty-nine specimens of *G. galloti* were captured in Tegueste, Tenerife (28°31′32.1″N 16°20′13.9″W) in July 2021. The sampling area was chosen due to the high prevalence of *A. cantonensis* in rats shown in previous studies (Martín-Carrillo *et al*., 2021) (Fig. 1). Lizards were captured alive with fall traps which were set in the morning and picked up in the afternoon. Once captured, the animals were brought to Instituto Universitario de Enfermedades Tropicales y Salud Pública de Canarias (IUETSPC) where they were anaesthetized using ketamine (Narkamon 100, Bioveta, Czech Republic) and dexmedetomidine (Dexdomitor 0.1, OrionPharma, Czech Republic) intramuscularly, and subsequently euthanized with T61 (MSD, Netherlands) intracardially. During dissection, squashed preparation of a part of liver tissue was examined for the presence of larvae by light microscopy. During microscopical examination, nematode larvae within granulomas were photographed using Leica ICC W camera and five of them were measured using LAS interactive measurement software. Remaining liver tissue was preserved in absolute molecular grade ethanol for the DNA isolation and in 4% formaldehyde. Parts of liver from lizards that presented larvae at microscopy and/or confirmed positive by qPCR were used for histopathological examination. Formalin-preserved liver samples were embedded in paraffin, cut and stained with haematoxylin–eosin. Processed samples were examined using Olympus BX53 microscope.

### DNA extraction, PCR and sequencing

Approximately 25 mg of liver tissue and proximal tail muscle tissue were cut into small pieces and used for the DNA extraction with DNEasy Blood&Tissue (Qiagen, Germany) extraction kit with modification optimized for L3 larvae of *A. cantonensis*, when the pre-lyse phase was extended overnight and 25 *μ*L instead of 20 *μ*L of proteinase K was used.

All 39 liver and 36 tail musculature samples were examined for the presence of *A. cantonensis* DNA by a species-specific qPCR assay (Sears *et al*., 2021) on QuantStudio™ 1 Real-Time PCR System, ThermoFisher at IUETSPC. The assay was performed in a 20 *μ*L reaction using 6.2 *μ*L of PCR water, 10 *μ*L of 2× MasterMix (IDT Prime time gene expression master), 0.2 *μ*L of 10 *μ*m probe (PrimeTime Eco Probe 5′ 6-FAM/ZEN/3′ IBFQ, /56-FAM/ACA TGA AAC/ZEN/ACC TCA AAT GTG CTT CGA/3IABkFQ/), 0.8 *μ*L of each 10 *μ*m primer (forward: AAA CTG TTG CTT TCG AAG CTA TG and reverse: GCG CAA ATC TGA CGT TCT TG) and 2 *μ*L of DNA template. Thermocycling (40 cycles) was made with the following cycling conditions: 95°C for 20 s followed by 40°C for 1 s and 60°C for 20 s. As positive controls, DNA from an adult female and a single L3 larva of *A. cantonensis* extracted by the same method as samples were used. The assay was run in duplicate. Only amplification curves with Ct value under 35 were taken as positive. To obtain the *A. cantonensis* DNA sequences, conventional PCR was used for amplification of the internal transcribed spacer 2 (ITS-2), partial 18SrDNA and partial COI in qPCR-positive samples of liver; for primers and conditions (see Table 1). Amplicons were sent to Macrogen (Spain and the Netherlands), and resulting sequences were analysed manually using Geneious Prime 2021.0.1 (http://www.geneious.com) and MEGA X and compared with those available in GenBank using BLAST.

**Table 1.** Primers and conditions used in conventional PCR assays

Gene	Primer	Primer sequence	Conditions			
ITS2	NC1	5′-ACGTCTGGTTCAGGGTTGTT-3′	94°C	1 min	30×	Gasser *et al*. (1993)
	NC2	5′-TTAGTTTCTTTTCCTCCGCT-3′	58°C	1 min		
			72°C	1 min		
			72°C	5 min		
COI	COI F1	5′-GGTGATTATAATGTTTAATG-3′	95°C	1 min		Lv *et al*. (2017)
	COI R1	5′-CGTAGGAACCGCAATAAC-3′	95°C	15 s	40×	
			55°C	15 s		
			72°C	25 s		
			72°C	5 min		
18S	AngioF1	5′-ATCATAAACCTTTTTTCGAGTATCCAG-3′	95°C	5 min		Qvarnstrom *et al*. (2007)
	AngioR1	5′-TCTCGAGACAGCTCAGTCCCGG-3′	95°C	15 s	45×	
			65°C	15 s		
			72°C	1 min		
			72°C	10 min		

## Results

**Fig. 2.**
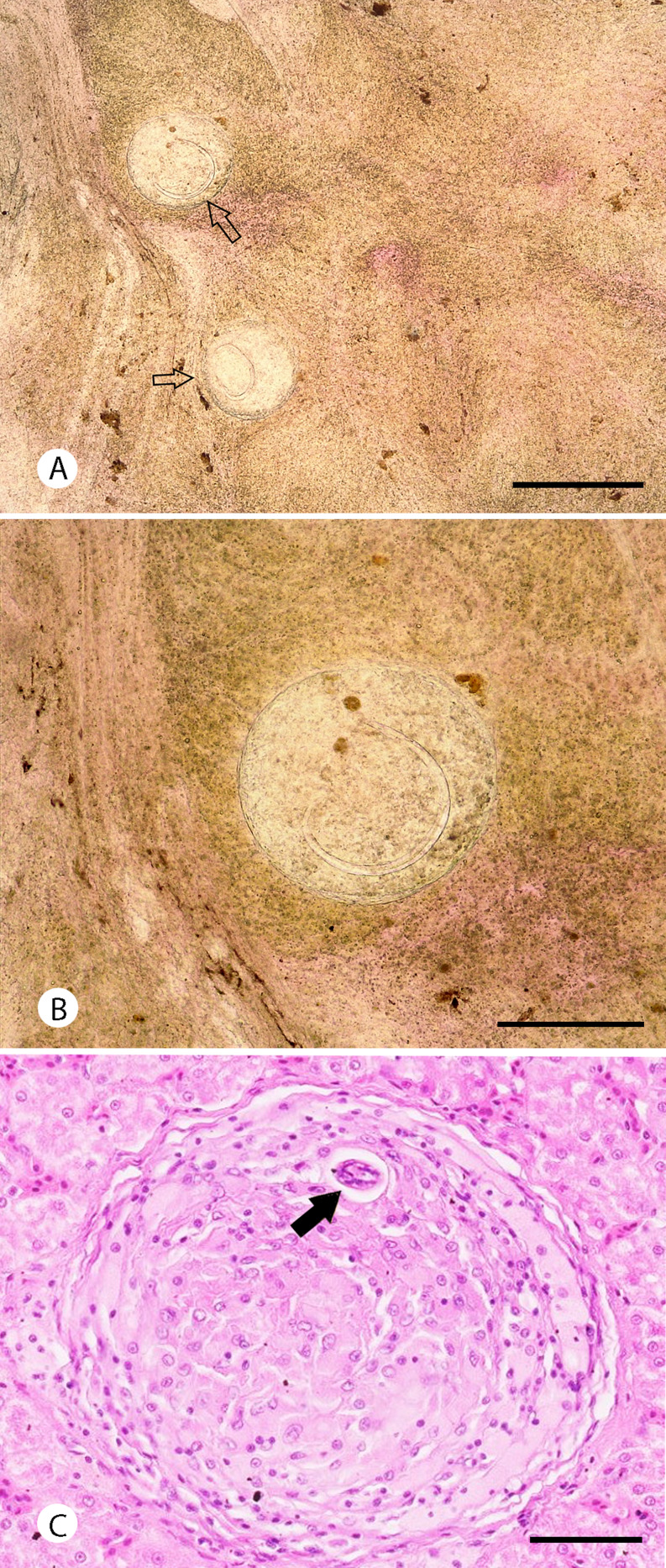
Metastrongylid larvae in the liver of *G. galloti*. Squashed preparation of the liver tissues (A, B); granuloma filled with inflammatory cells with visible cross-section through the larva (black arrow). Scale bars: 500 *μ*m (A), 250 *μ*m (B) and 50 *μ*m (C).

The microscopic examination showed eight out of 36 samples (22.2%) presenting granulomas with metastrongylid larvae inside (Fig. 2A and B). The length of measured larvae was 573.9 ± 23.7 *μ*m (95%). Histopathological examination of a liver sample (with the presence of *A. cantonensis* confirmed by qPCR) showed granulomas containing inflammatory cells such as macrophages and lymphocytes, with transversal sections through the nematode larvae (Fig. 2C).

qPCR results confirmed the presence of *A. cantonensis* DNA in liver and tail muscular tissue of *G. galloti*. Ten out of 39 (25.6%) liver samples and seven out of 36 (19.4%) tail samples tested positive by qPCR with Ct value under 35 cycles. Conventional PCR was only performed in the positive samples by qPCR and confirmed the presence of *A. cantonensis* DNA in several of these samples as well. Positivity in tail muscles corresponded with DNA detection in the liver in six samples, one sample from the tail showed positive, even though the liver sample from the same individual was negative.

However, not all microscopically positive samples were confirmed positive by qPCR. Five samples where metastrongylid larvae were observed microscopically were negative by *A. cantonensis*-specific qPCR and, on the contrary, seven samples in which we did not observe larvae microscopically were positive for *A. cantonensis* DNA when tested by qPCR. Differences between microscopical and molecular analysis are summarized in Table 2.

**Table 2.** Comparison of results of microscopy of squashed liver samples and molecular methods

Sample no.	Microscopy	qPCR liver	qPCR tail muscle	ITS2 sequence (BLAST)	COI sequence (BLAST)	18S sequence (BLAST)
GG1–6	Negative	Negative	Negative	–	–	–
GG7	Positive	Negative	Positive	–	–	–
GG8	Negative	Positive	Positive	–	–	–
GG9	Positive	Negative	Negative	–	–	–
GG10	Positive	Positive	Negative	100% *A. cantonensis* (AB700700.1)	–	–
GG11	Negative	Positive	Positive	100% *A. cantonensis* (AB700700.1)	98.42% *A. cantonensis* (MK570629.1)	100% *A. cantonensis* (AY295804.1)
GG12	Negative	Negative	Negative	–	–	–
GG13	Negative	Positive	Negative	99.6% *A. cantonensis* (AB700700.1)	–	–
GG14–20	Negative	Negative	Negative	–	–	–
GG21	Negative	Positive	Positive	–	–	–
GG22	Negative	Negative	Negative	–	–	–
GG23	Positive	Negative	Negative	–	–	–
GG24	Positive	Negative	Negative	–	–	–
GG25–26	Negative	Negative	Negative	–	–	–
GG27	Positive	Positive	Positive	–	–	–
GG28	Negative	Positive	Positive	–	–	–
GG29–30	Negative	Negative	Negative	–	–	–
GG31	Positive	Positive	Positive	99.71% *A. cantonensis* (AB700700.1)	100% *A. cantonensis* (MK570629.1)	100% *A. cantonensis* (AY295804.1)
GG32	Negative	Negative	Negative	–	–	–
GG33	Negative	Positive	Negative	–	–	–
GG34	Negative	Negative	Negative	–	–	–
GG35	Positive	Negative	Negative	–	–	–
GG36	Negative	Negative	Negative	–	–	–
GG37	Negative	Positive	–	–	–	–
GG38–39	Negative	Negative	–	–	–	–

Differences in results of microscopy and molecular analysis may indicate not only that qPCR (Sears *et al*., 2021) is more sensitive and specific diagnostic method than mere microscopical examination and conventional PCR, but also that possibly other metastrongylid species found *G. galloti* as a suitable paratenic host.

*Note*: The positive result of microscopy means just microscopical observation of nematode larvae, not confirmation of *A. cantonensis* presence.

Two partial COI sequences of length 405 and 635 bp were obtained. When compared to those available in GenBank, these partial sequences were 98.42 and 100% identical to the *A. cantonensis* lineage TEN.1 from the Tenerife, clustering into the clade 2 with all the invasive lineages, as reviewed by Červená *et al*. (2019). Four partial ITS-2 sequences (287–373 bp) and two partial 18S sequences (518 and 619 bp) were obtained, all showing a high level of identity with *A. cantonensis* (99.6–100%, see Table 2).

## Discussion

*Angiostrongylus cantonensis* is well-known for its complex circulation in food webs (Wallace and Rosen, 1967; Alicata, 1991; Paredes-Esquivel *et al*., 2019). Its life cycle involves rats as definitive hosts, aquatic and terrestrial gastropods as intermediate hosts and a range of poikilotherm paratenic hosts (Wallace and Rosen, 1967; Ash, 1968; Radomyos *et al*., 1994). The parasite has been found naturally infecting monitor lizards *Varanus bengalensis*. These apex predators accumulate high numbers of infective larvae, predominantly present in the liver, much less so in skeletal muscles and the intestine (Radomyos *et al*., 1994). Infectivity of reptile-derived larvae for a definitive host was confirmed experimentally (Radomyos *et al*., 1994).

Abundance of *G. galloti* in Tenerife and their co-occurrence with both invasive rat species makes them ideal paratenic hosts for *A. cantonensis*. Indeed, we found common presence of metastrongylid larvae in the liver of *G. galloti* microscopically and confirmed the presence of *A. cantonensis* by species-specific qPCR and ITS2, COI and 18S sequencing. Considering high sensitivity and specificity of the qPCR assay used (Sears *et al*., 2021), the observed partial discrepancy between microscopy and qPCR data suggests the co-occurrence of *A. cantonensis* larvae together with those of other metastrongylid nematodes. These undetermined larvae might belong to *Angiostrongylus vasorum*, *Aelurostrongylus abstrusus* or *Crenosoma striatum*, which were previously reported from the Canary archipelago (Sánchez Vicente, 2013; Segeritz *et al*., 2021). Further research is needed to clarify the role of endemic lizards in the life cycle of metastrongylids in Macaronesian terrestrial ecosystems and the potential impact of these infections on the fitness of lizards with high ecological value.

*Gallotia galloti* is a dominant reptile in Tenerife and its altitudinal distribution ranges from sea level until more than 3000 m (Báez, 2002). As a result, the diet of this lizard species varies according to ecosystem type, climate and season; however, plant material (leaves, flowers and fruits) always represents its major part (Valido and Nogales, 1994, 2003). Terrestrial gastropods were demonstrated as a minor part of the diet of *G. galloti* during the colder months (Valido *et al*., 2003).

*Gallotia* spp. play an important role in the island ecology, not only as seed dispersers, but also as part of the diet of native and alien carnivores in the archipelago which can expose these predators to the infective larvae of *A. cantonensis*. In birds, *G. galloti* remnants were identified in 14.2 and 26.1% of samples from Canarian kestrels *Falco tinnunculus canariensis* and common ravens *Corvus corax*, respectively (Nogales and Hernández, 1994; Molina-Borja and Bischoff, 1998; Carrillo *et al*., 2017). Also, the Southern grey shrike *Lanius meridionalis* feeds on lizards (Padilla *et al*., 2007). In the case of mammals, *G. galloti* is an important part of the diet of feral cats (*Felis silvestris catus*), being present in 74.2% of total fecal samples analysed in some areas of Tenerife (Nogales *et al*., 1990; Medina and Nogales, 2008). Rarely *G. galloti* has been also reported as a prey for the common frog (*Pelophylax perezi*), red-backed shrike (*Lanius collurio*) and the sparrowhawk (*Accipiter nisus*) (Nogales *et al*., 1988; Barone and Trujillo, 1997; Barone *et al*., 2006).

The high infection rate of *G. galloti* with *A. cantonensis* implies the possibility of its transmission to accidental avian hosts in which it could potentially cause neurological diseases (Monks *et al*., 2005; Ma *et al*., 2013). Since a fatal case of cerebral infection due to *A. cantonensis* was previously described in a pygmy falcon (*Polihierax semitorquatus*) implying a gecko as the probable cause of infection (Burns *et al*., 2014), it is likely that infection of Canarian kestrels can impact on their fitness in localities with high prevalence of the infection in lizards. It has been suggested that invasive rats may feed on juveniles and eggs of *Gallotia* spp. (Pleguezuelos *et al*., 2002). However, as there is a lack of data regarding the amount of lizards eaten by rats, the extent in which infected *Gallotia* contribute to the infection of rats as definitive hosts remains unclear.

Recently described intermediesis, i.e. transmission of metastrongylid L3 larvae between two susceptible hosts at the same trophic level (i.e. gastropods, Colella *et al*., 2015; Modrý *et al*., 2020), opens a question of transmission of *A. cantonensis* among lizards by cannibalism. Such a unique mode of transmission has been proven in dihomoxenous apicomplexan protists of *Gallotia*, such as *Sarcocystis gallotiae* (Matuschka and Bannert, 1987).

Although the date and way of arrival of *A. cantonensis* to Tenerife is still uncertain, the high prevalence in rats and its presence among endemic intermediate and paratenic hosts (*P. lamarckii*, *G. galloti*) evidence that the parasite is firmly established in local ecosystem (Martin-Alonso *et al*., 2015; Martín-Carrillo *et al*., 2021). *Gallotia galloti* is endemic to Tenerife and La Palma; however, its accidental introduction to other islands has been reported (Rodríguez-Domínguez and Ruiz-Caballero, 1998; Mateo-Miras and Pérez-Mellado, 2005). Dispersal of *G. galloti* outside of its original range of distribution might increase the risk of spread of *A. cantonensis* to new islands.
